# Buttocks or Breasts? Identifying Latent Subgroups in Male Preferences for Female Bodily Features

**DOI:** 10.1177/14747049261452897

**Published:** 2026-06-17

**Authors:** Mehmet Mehmetoglu

**Affiliations:** 1 Department of Psychology, 8018 Norwegian University of Science and Technology, Trondheim, Norway

**Keywords:** buttocks, breasts, physical, attractiveness, male preference, bodily, LPA

## Abstract

Among the various physical traits that serve as secondary sexual characteristics, breasts and buttocks are visually conspicuous cues used in male mate evaluation because they signal youth, fertility, developmental stability, and nutritional status. Although these features have been extensively studied, less is known about the extent to which men differ in the relative importance they place on each trait. Latent Profile Analysis (LPA) identified distinct subgroups of Norwegian men (*N = *395) based on how much importance they placed on breasts and buttocks in their evaluations of romantic partners. A four-class solution emerged, comprising Highly Body-Oriented (53%), Moderately Body-Oriented (20%), Buttocks-Oriented (18%), and Body-Unoriented (9%) subgroups. Predictors such as egalitarianism, materialism, and preferences for sexiness were, as expected, significantly associated with class membership, providing support for the validity of the typology. Notably, a consistent buttocks-oriented class emerged across all LPA solutions, whereas no distinct breasts-oriented group was found, as ratings of breasts persistently co-occurred with high ratings of buttocks, suggesting that breasts do not function as a primary preference in isolation. These findings reveal that buttocks may occupy a more central and stable role than breasts in male mate preferences, offering novel insights into the structure of physical attraction and heterogeneity in the male mating mind.

## Introduction

Mate choice is typically a lifelong and future-shaping decision, making it one of the most critical choices an individual can make. Since their evolutionary origins, humans have relied on evolved mate selection criteria including physical, sociocultural, and cognitive traits to make adaptive choices. Among these, men have historically placed greater emphasis on facial and bodily physical features when assessing a potential mate. This preference for visual and bodily cues remains strong even today (Buss, 1989), and is evident across both short-term and long-term mating strategies, although additional traits such as kindness and intelligence tend to gain importance in long-term contexts (N. P. Li & Kenrick, 2006).

Physical attractiveness plays a significant role in male mate choice across diverse cultural contexts (see Gangestad & Scheyd, 2005) much like in many other species. Human males, too, rely on physical features as cues to underlying indicators of mate quality, such as immune competence, developmental stability, genetic fitness, youth, fertility, and reproductive value (Thornhill & Gangestad, 1999). These physical traits are typically categorized into facial features (e.g., hair, eyes, skin texture) and bodily features (e.g., waist-to-hip ratio [WHR], breast size), which together contribute to an individual's physical attractiveness. Each of these features has been the focus of extensive empirical research within an evolutionary psychology framework.

With regard to facial features, for example, Hinsz et al. (2001) suggest that both *hair* length and quality serve as visible indicators of youth and health. Kościński (2012) reports that reduced *eyebrow* thickness in women is linked to greater sexual attractiveness. Peshek et al. (2011) find that both male and female observers rate faces with a distinct *limbal ring* as more attractive.

Pazhoohi and Kingstone (2023) show that *eyelash* length enhances attractiveness. Claes et al. (2015) argue that bilateral *ear* symmetry may primarily be driven by sexual selection. Van Zijl et al. (2020) demonstrate that a mathematically averaged composite *nose* is judged more attractive than the individual noses it comprises. Alais et al. (2025) highlight *lip* size as a key factor in shaping perceptions of attractiveness. Hendrie and Brewer (2012) find that irregular *tooth* spacing and yellow discoloration significantly reduce attractiveness. According to Little et al. (2011), *jaw* morphology reflects underlying processes of masculinisation and feminisation. Jones et al. (2004) provide evidence for a positive link between the apparent health of *facial skin* and male facial attractiveness ratings. A recent study (Kleisner et al., 2024) further shows that facial attractiveness is primarily associated with average facial shape and femininity, whereas facial symmetry and male facial masculinity have little or no effect. Finally, Li et al. (2025) show that people generally prefer younger-looking faces, but this preference is unrelated to parental age at birth.

Turning to bodily features, Pazhoohi et al. (2023) report that both male and female participants find a higher shoulder-to-hip ratio (SHR) more attractive in both sexes. Singh (1993) provides compelling evidence that body fat distribution, as measured by WHR, is systematically linked to indicators of youthfulness. Gynoid fat, which accumulates primarily in the *thighs*, is therefore generally perceived as physically attractive (Singh, 1994).

Youthful-looking *hands* are also considered significantly more desirable in women (Kościński, 2012). Fessler et al. (2005) suggest that smaller *feet* enhance perceptions of female attractiveness. Similarly, Sorokowski and Pawlowski (2008) found that figures with shorter-than-average *legs* were consistently rated as less attractive by both male and female participants. According to Tovée et al. (1999), *body mass* index (BMI) is a stronger predictor of female body attractiveness than WHR. Finally, Azevedo (2021) reports that men prefer women without visible *body hair*, regardless of whether it appears on the legs or underarms.

### Breasts and Buttocks

Among the many physical traits that function as secondary sexual characteristics, breasts and buttocks are visually conspicuous cues relevant to reproductive value and mate evaluation. As Cant notes, humans have been interested in breasts and buttocks, in one way or another, for a long time, as evidenced by the Venus figurines from Upper Paleolithic Europe (Cant, 1981, p. 199). These features have consequently attracted significant scholarly attention for their presumed roles in human evolution and sexual selection.

Starting with breasts, human females are unique in developing enlarged, fat-rich breasts at puberty, well before pregnancy or lactation, unlike other primates, in which breast development typically occurs only during the first pregnancy (Cant, 1981). According to Miller (2000), this trait likely evolved through sexual selection to signal three key aspects of female quality: youth and fertility, developmental stability, and nutritional status.

First, Miller (2000) suggests that hominid males back in the Pleistocene probably favoured younger women for their higher fertility, and accordingly, any indicator of youth, such as large and firm breasts, would tend to be favored by males. Second, it is possible that bipedalism made breasts a useful potential cue of developmental stability for male mate choice, in that once men started paying attention to the symmetry of breast development, high-fitness women could better display that symmetry by evolving large breasts (Miller, 2000). Third, in the Pleistocene, starvation posed a greater threat than overeating, and females who concentrated fat in the breasts (and buttocks) could both attract male interest and avoid overheating, unlike if the fat were evenly distributed across the body (Miller, 2000).

As such, numerous studies have investigated how breast morphology, particularly size, firmness, and symmetry, influences the perceived physical attractiveness of human females. For example, Dixson et al. (2011) found that men from New Zealand, Papua New Guinea, and Samoa consistently preferred medium and large breast sizes over smaller ones. Similarly, Pazhoohi et al. (2020) reported that firmer breasts significantly enhanced attractiveness ratings. Lastly, Singh (1995) found that college males judged symmetrical breasts to be the most attractive.

Turning to buttocks, the shift to upright walking around 4.2 million years ago in our hominid ancestors led to a reengineering of the legs and buttocks, resulting in the evolution of much larger and stronger muscles designed to power the legs backward and propel the body forward—muscles that give the human buttocks their distinctive rounded shape, unique to our species, with human females evolving even larger fat deposits in this region as part of that adaptation (Miller, 2000). Miller (2000) suggests that, like breasts, buttocks evolved through male mate choice to signal youth and fertility, sufficient fat reserves, and possibly developmental stability.

Buttocks morphology involves two key components: shape and size. Regarding shape, two empirical studies have highlighted its importance in perceived attractiveness. The first, by Singh (1993), found that men perceive women with a low WHR of approximately 0*.*70 as more attractive, as higher WHR values are associated with greater difficulty in becoming pregnant, thereby linking lower WHR to youth and fertility. The second, relatively more recent study by Lewis et al. (2015), showed that men prefer greater vertebral wedging, which produces a more pronounced lumbar curvature, optimally around 45*.*5°. This curvature not only enhances visual appeal but also reduces the biomechanical challenges associated with pregnancy, making it both aesthetically appealing and evolutionarily advantageous.

The study by Lewis et al. (2015) further indicates that perceived attractiveness depends not merely on the degree of buttock protrusion, but on its underlying anatomical cause: protrusion produced by spinal curvature was preferred over protrusion produced by increased soft-tissue mass. Thus, the findings point to the importance of morphological structure rather than sheer volume. Evidence concerning buttocks size addresses a different issue and appears more variable. For example, Jones (1996) reports cross-cultural differences, with Brazilian men tending to prefer larger buttocks, whereas men from the USA and Russia show a preference for smaller ones. Similarly, Furnham and Swami (2007) found that buttocks size was not a significant determinant of perceived female attractiveness. These findings therefore do not contradict the structural account, but instead indicate variability in size preferences across populations.

Although there is ongoing debate and research about which specific features, such as size, shape, and symmetry, of the buttocks and breasts contribute most to female physical attractiveness, there is general consensus that these two traits attract male attention. This is further supported by two separate eye-tracking studies: one by Dixson et al. (2011), which highlights the visual attention directed toward breasts, and a more recent study by Zeng et al. (2024), which demonstrates that buttocks similarly capture male attention and influence perceptions of female attractiveness.

It is well established that buttocks and breasts are key features of female physical attractiveness and are salient to many men across diverse cultural contexts. Experimental Studies show that variation in breast size and hip–buttock morphology reliably influences men's attractiveness judgments (Dixson et al., 2011; Singh & Young, 1995). Moreover, similar tendencies have been documented in non-Western and small-scale societies, supporting their cross-cultural salience (Marlowe et al., 2005). However, can men be meaningfully distinguished based on the strength of their preference for one of these two biologically significant traits? If we were to assume that both traits are equally valued by all men, population-level averages could be used to estimate their relative salience and identify which trait men tend to favor. However, as with most preferences, we should expect variability around these averages, reflecting heterogeneity in male preferences.

This heterogeneity can be examined through latent profile analysis (LPA), a statistical method designed to uncover unobserved subgroups within a population based on patterns across a certain set of observed variables (Spurk et al., 2020). Such profiling allows for a more nuanced understanding of men's mating psychology and behavior by identifying distinct subgroups according to their trait preferences. To the best of the author's knowledge, no study has investigated this issue in a comprehensive and targeted manner. The only study that approaches this line of inquiry is that of Dagnino et al. (2012), which, using eye-tracking, found that more men focused on buttocks than on breasts when assessing female attractiveness, suggesting that buttocks may exert a stronger influence.

**Figure 1.**
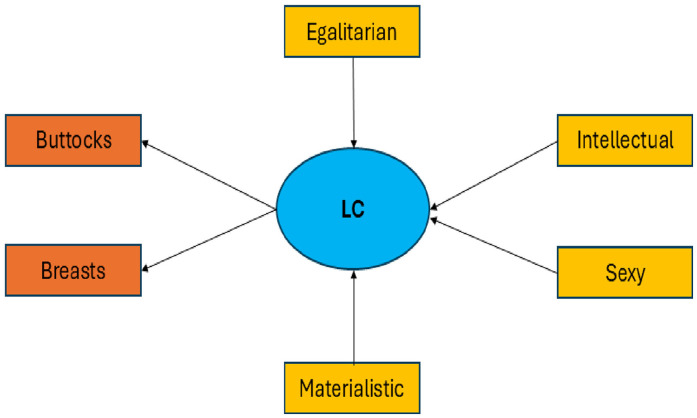
The study's LPA/LCA model (with two class indicators and four predictors).

The present study aims to address this gap, using our LPA framework (see Figure 1). We conduct the analysis in Norway, a Western, Educated, Industrialized, Rich, and Democratic (WEIRD) society, which according to Maryanski (2010) is particularly well-suited for evolutionary psychology research. Norway represents a social context characterized by high gender equality, strong welfare provisions, and relatively high mate-choice autonomy. These conditions may reduce some of the material constraints that can influence mate preferences in more resource-constrained environments. As a result, variation in preferences may be more readily observable as individual differences within a shared cultural context. Importantly, this does not imply that preferences are entirely free from social or structural influences; rather, the LPA aims to capture heterogeneity in preferences within a context where certain constraints on mate choice are comparatively weaker.

## Method

### Procedure

The data collection was conducted in the spring of 2023 using a questionnaire. It took place in Norway and employed *Nettskjema*, a Norwegian online tool for creating and distributing electronic surveys and consent forms, developed and operated by the University of Oslo. An anonymised link to the survey was distributed via social media and on a university campus by a group of students, resulting in a total of 1154 respondents, of whom 6 did not report their gender and 753 identified as women, yielding a final subsample of 395 Norwegian men. This was subsequently used in the study's LPA.

### Measures

Respondents were asked, “How important would you consider these physical qualities (buttocks and breasts) in a partner?,” and rated their importance on a 5-point scale ranging from 1 (not at all important) to 5 (very important). These variables formed the basis for the LPA depicted in Figure 1. In addition, four variables were included as external predictors for validation purposes, based on the assumption that they would meaningfully differentiate the latent classes identified through the LPA. The first two captured respondents’ egalitarian orientation and materialistic orientation. Egalitarianism was assessed with the item “Everyone should have an equal chance and an equal say,” while materialism was measured with the item “I would like to possess things/achievements that impress others.” The remaining two variables measured the importance respondents placed on sexiness and intellectuality, respectively, as general mate choice criteria. All six variables were rated on a scale ranging from 1 to 5.

### Statistical Analysis

To capture the anticipated heterogeneity in the data, specifically the presence of latent profiles or classes, a LPA was conducted using Mplus Version 8.10 (L. K. Muthén & Muthén, 1998–2017), facilitated through the MplusAutomation package (Hallquist & Wiley, 2018) in R. Models specifying one to six latent classes were estimated. Following estimation, the four-class solution was selected and labeled based on the pattern of class-specific means and theoretical interpretability. The LPA was accompanied by a validation procedure in which the associations between latent class membership and four external predictors were assessed using Vermunt's three-step approach (Vermunt, 2010), implemented via the R3STEP procedure in Mplus. This method examines the relationships between latent classes and external predictors while preserving the class structure identified in the initial LPA. The procedure consists of three steps: (1) estimating the latent class model using only the indicator variables; (2) assigning individuals to their most likely class based on posterior probabilities; and (3) regressing class membership on external predictors while adjusting for classification uncertainty introduced in the second step (Asparouhov & Muthén, 2014). The resulting estimates correspond to a multinomial logistic regression model. Incidentally, although the study was exploratory, we chose LPA because it provides a model-based, person-centred approach that is better suited to identifying unobserved subgroups than heuristic clustering methods (e.g., k-means). Unlike distance-based clustering, LPA estimates profiles within a probabilistic framework, allowing classification uncertainty to be quantified, measurement error to be incorporated, and model fit to be evaluated using formal information criteria (e.g., AIC, BIC, and entropy). This also enabled us to compare alternative profile solutions systematically and to use posterior membership probabilities.

### Sample Characteristics

The sample consisted of 395 Norwegian men. Participants were relatively young (*M* = 22.76 years, SD = 3.27), with ages ranging from 18 to 55, although the distribution was strongly concentrated in the early twenties. Most respondents were between 21 and 25 years old, and more than half of the sample fell within the 21–23 age range alone, indicating that the data primarily represent young adult men rather than a broadly age-diverse population. Regarding relationship status, 37.5% of the respondents reported that they were currently in a romantic relationship (*n = *148), whereas 62.5% indicated that they were not (*n = *247). Thus, the sample was predominantly single at the time of participation. Together, these characteristics suggest that the dataset mainly reflects mating preferences among young adult men, many of whom were not in an ongoing relationship.

## Results

### Descriptives and Correlations

As shown in Table 1, among the two physical features used as indicators in the LPA analysis, Norwegian men rated buttocks higher (*M = *3.91, SD = 0.97) than breasts (*M = *3.37, SD = 1.05). Regarding the general mate choice criteria, participants rated “sexy” (*M = *3.84, SD = 0.93) and “intellectual” (*M = *3.78, SD = 0.94) similarly. The remaining two predictors captured attitudinal orientations: egalitarian attitudes received higher endorsement (*M = *3.97, SD = 1.02) than materialistic attitudes (*M = *2.88, SD = 1.15), suggesting a stronger preference for egalitarianism among Norwegian men. All variables were assessed on a 5-point scale ranging from 1 to 5. Due to item-level missingness, sample sizes varied slightly across variables, ranging from 338 (breasts) to 390 (materialistic).

**Table 1.** Descriptive Statistics for the Latent Class Indicators and Predictors.

Variable	n	Mean	SD	Min	Max
Buttocks	358	3.91	0.97	1	5
Breasts	338	3.37	1.05	1	5
Egalitarian	388	3.97	1.02	1	5
Materialistic	390	2.88	1.15	1	5
Sexy	357	3.84	0.93	1	5
Intellectual	358	3.78	0.94	1	5

Furthermore, the Pearson correlations between the latent class indicators and predictors are presented in Table 2. As expected, ratings of the two reproductive features (buttocks and breasts) were positively correlated (*r =* .41, *p < *.01). Materialistic orientation was positively associated with ratings of both buttocks (*r =* .28, *p < *.01) and breasts (*r =* .14, *p < *.05), as well as with the general preference for sexiness as a mate choice criterion (*r =* .22, *p < *.01). Preference for sexiness was also positively associated with ratings of buttocks (*r =* .45, *p < *.01) and breasts (*r =* .40, *p < *.01), as expected. In contrast, egalitarian orientation was negatively correlated with ratings of both buttocks (*r =* –.09, *p < *.10) and breasts (*r =* –.10, *p < *.10). Finally, preference for intellectuality as a mate choice criterion was positively correlated with preference for sexiness (*r =* .23, *p < *.01), but was not significantly associated with any of the other variables. Finally, and reflecting prior assumptions, materialistic and egalitarian orientations were negatively associated with each other (*r =* –.10, *p < *.10).

**Table 2.** Pearson Correlation Matrix of the Latent Class Indicators and Predictors.

	Buttocks	Breasts	Egalitarian	Materialistic	Sexy	Intellectual
Buttocks breasts	0.41***					
Egalitarian	−0.09*	−0.10*				
Materialistic	0.28***	0.14**	−0.10*			
Sexy	0.45***	0.40***	−0.01	0.22***		
Intellectual	0.04	0.07	−0.05	−0.00	0.23***	

*p < .10; **p < .05; ***p < .01.

### Choice of the 4-Class Solution

Table 3 presents the fit indices for the six-class solutions. Based primarily on the sample-size adjusted BIC (aBIC) and the bootstrap likelihood ratio test (BLRT) *p*-values (*p < *.10), each successive model demonstrated improved fit over the preceding one. However, the five-class and six-class solutions exhibited signs of local maxima, raising concerns about their stability. Consequently, we selected the four-class model, which did not show such estimation issues and offered a well-fitting, stable, and theoretically meaningful solution without convergence problems. Moreover, the average posterior probabilities (AvePP) for individuals’ most likely class assignments all exceeded 0.70, indicating well-separated latent classes (Nylund-Gibson & Choi, 2018). In addition, while the four-class model's entropy was of medium magnitude (Clark, 2010), it still remained within an acceptable range. It is also generally discouraged to override superior fit indices such as aBIC and BLRT *p*-values based solely on entropy values (B. Muthén, 2007). Finally, the smallest class in the four-class solution comprised 9% of the sample, which does not represent a critically small proportion (Vanslambrouck et al., 2019).

**Table 3.** Fit Statistics for 1 Through 6 Latent Class Solutions.

Title	Parameters	LL	BIC	aBIC	BLRT_PValue	Entropy	Smallest_class
1_Class	4	−992.033	2007.587	1994.897	NA	NA	NA
2_Class	7	−942.006	1925.175	1902.968	0.000	0.854	NA
3_Class	10	−930.620	1920.046	1888.321	0.000	0.781	NA
4_Class	13	−922.614	1921.676	1880.433	0.006	0.668	NA
5_Class	16	−659.602	1413.292	1362.532	0.070	0.924	NA
6_Class	19	−655.019	1421.767	1361.490	0.099	0.885	NA

Note: LL = log-likelihood; BIC = Bayesian Information Criterion; aBIC = sample-size adjusted BIC; BLRT_PValue = Bootstrap Likelihood Ratio Test *p*-value; entropy = classification accuracy; Smallest_class = proportion in the smallest latent class.

### Profiling the 4 Classes

**Figure 2.**
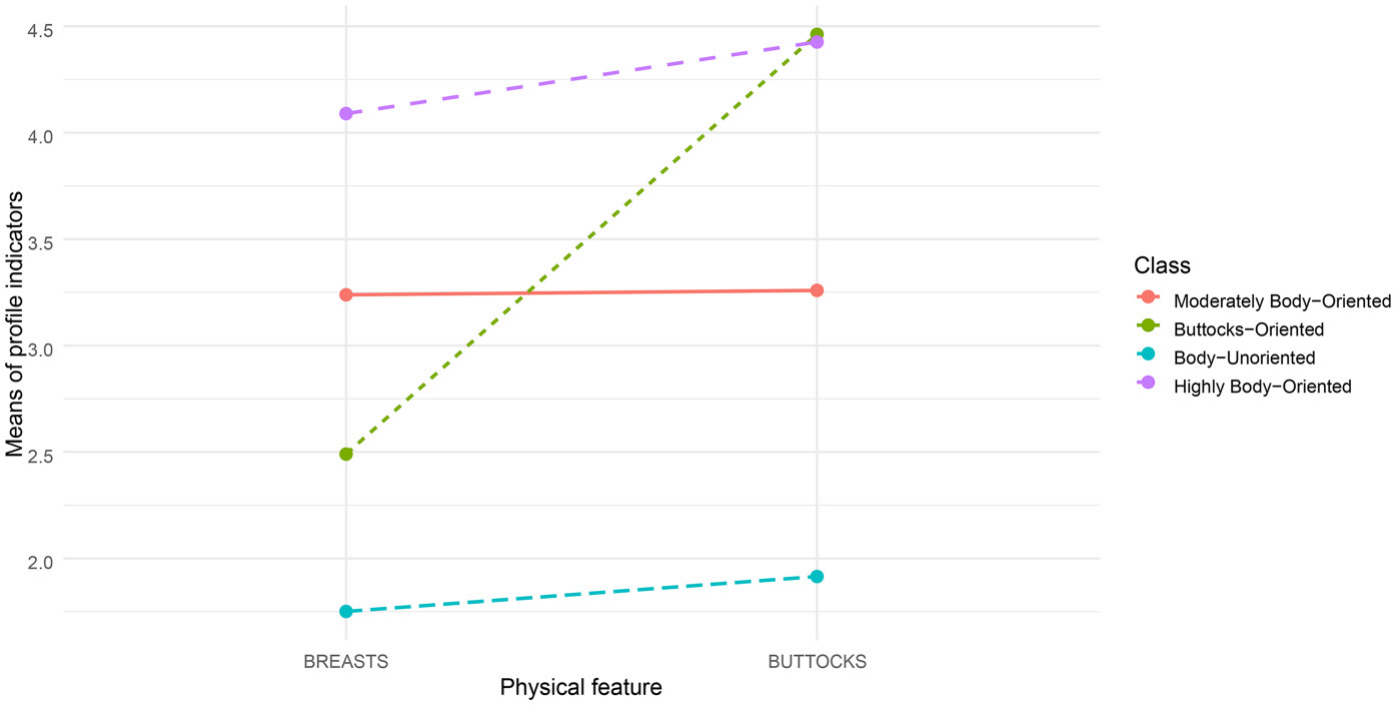
Depiction of the four latent classes defined by the pattern of means on buttocks and breasts.

Figure 2 displays the mean ratings of physical features (breasts and buttocks) across the four latent classes. As illustrated, the Highly Body-Oriented class reported consistently high ratings for both breasts (*M = *4.1) and buttocks (*M = *4.4), indicating a strong emphasis on physical traits in mate preferences. This class comprised 53% of the sample. The Moderately Body-Oriented class showed moderate and nearly equal interest in both features (*M = *3.2), reflecting a balanced but less intense focus on physicality, and represented 20% of the sample. In contrast, the Buttocks-Oriented class demonstrated a clear preference for buttocks (*M = *4.5) over breasts (*M = *2.5), suggesting a specific feature-oriented pattern; this group accounted for 18% of participants. Finally, the Body-Unoriented class gave the lowest ratings to both breasts (*M = *1.8) and buttocks (*M = *1.9), implying that physical traits play a minimal role in their mate selection criteria. This class comprised 9% of the sample. These profiles underscore the heterogeneity in physical preferences and highlight distinct patterns of body orientation across individuals in the sample.

### Predictors of the 4 Classes

As we see in Table 4, using the body-unoriented class as the reference category, we examined how individual differences in egalitarian and materialistic attitudes, and preference for sexiness and intellectuality predicted the latent class membership. Preference for sexiness significantly increased the likelihood of being in any of the three body-oriented classes. Specifically, higher sexiness scores were associated with greater probability of belonging to the moderately body-oriented (Estimate = 1.06, SE = 0.31, *p = *.01), buttocks-oriented (Estimate = 1.50, SE = 0.36, *p < *.01), and highly body-oriented (Estimate = 1.79, SE = 0.44, *p < *.01) groups.

**Table 4.** Key Predictors of the Latent Class Membership.

Predictor	Statistic	Moderately_Body_Oriented	Buttocks_Oriented	Highly_Body_Oriented
Egalitarianism	Estimate	−0.776	−0.742	−1.004
	SE	0.401	0.467	0.421
	*p*-value	.053*	.113	.017**
Intellectual	Estimate	−0.229	−0.339	−0.005
	SE	0.256	0.332	0.279
	*p*-value	.371	.307	.987
Material	Estimate	0.116	0.618	0.630
	SE	0.303	0.340	0.293
	*p*-value	.702	.069*	.031**
Sexy	Estimate	1.061	1.503	1.794
	SE	0.308	0.355	0.443
	*p*-value	.001***	.000***	.000***

Note: Reference category is body unoriented.

***p < .01, **p < .05, *p < .10.

Materialism also positively predicted membership in the buttocks-oriented (Estimate = 0.62, SE = 0.34, *p = *.069) and highly body-oriented (Estimate = 0.63, SE = 0.29, *p = *.031) classes, though not in the moderately body-oriented group. Egalitarianism was negatively associated with membership in all three body-oriented classes. This relationship was statistically significant for the highly body-oriented group (Estimate = −1.00, SE = 0.42, *p = *.017), marginally significant for the moderately body-oriented group (Estimate = −0.78, SE = 0.40, *p = *.053), and suggestive, though not statistically significant, for the buttocks-oriented group (Estimate = −0.74, SE = 0.47, *p = *.113). In contrast, intellectual orientation did not significantly predict membership in any of the latent classes.

Logit coefficients (*β*) were interpreted as percentage changes in odds using (*e^β^* −1) × 100. A one-unit increase in sexiness increased the odds of belonging to the moderately by 189%, buttocks-oriented classes by 349%, and highly body-oriented classes by 501%. Materialism increased the odds of the buttocks-oriented classes by 86% and highly body-oriented classes by 88%, whereas egalitarianism reduced the odds of the highly body-oriented class by 63% and the odds of the moderately body-oriented class by 54%; intellectuality showed no association. The remaining logit coefficients were small and statistically non-significant. When all four predictors were entered simultaneously to predict class membership, the model accounted for approximately 20% (Cragg and Uhler's pseudo-*R*^2^) of the variation in class assignment.

As shown in Table 5, to further assess the mean differences in the two profile indicators across the four classes, we estimated a multivariate regression model in which both indicators were treated as dependent variables and class membership served as the grouping variable within a single omnibus model. The analysis was conducted within the structural equation modeling (SEM) framework using the lavaan package in R (Rosseel, 2012). All six possible pairwise comparisons yielded statistically significant differences in means, reinforcing the robustness of the observed distinctions between classes.

**Table 5.** Pairwise Comparisons Among the Four Classes.

Comparison	buttocks_est	Se1	P1	breasts_est	Se2	p2
Buttocks-Oriented vs. Moderately Body-Oriented	1.677	0.073	<.001	−0.629	0.182	<.001
Body-Unoriented vs. Moderately Body-Oriented	−1.156	0.091	<.001	−1.389	0.225	<.001
Highly Body-Oriented vs. Moderately Body-Oriented	1.337	0.059	<.001	0.760	0.147	<.001
Body-Unoriented vs. Buttocks-Oriented	−2.833	0.092	<.001	−0.760	0.229	<.001
Highly Body-Oriented vs. Buttocks-Oriented	−0.340	0.061	<.001	1.389	0.152	<.001
Highly Body-Oriented vs. Body-Unoriented	2.493	0.081	<.001	2.149	0.202	<.001

**Figure 3.**
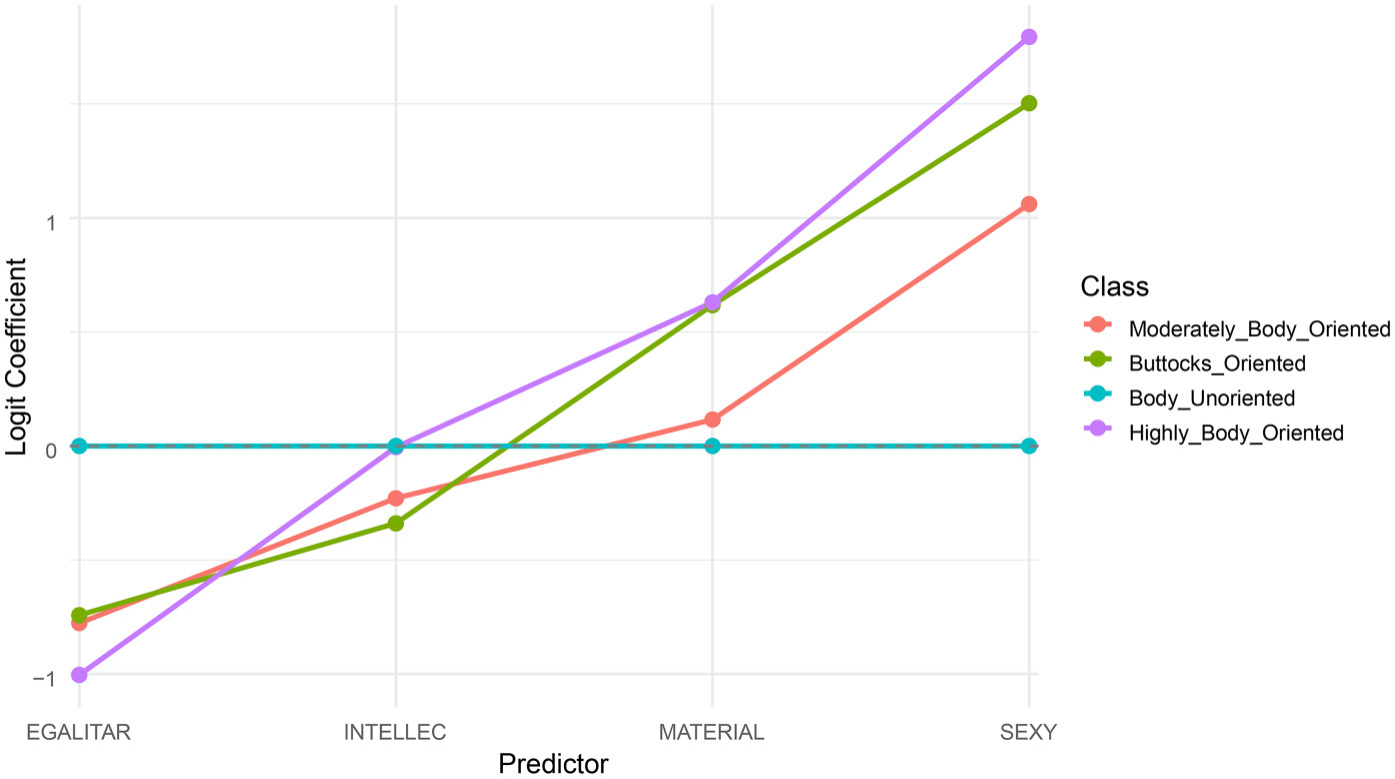
Visualising the relationship between the predictors and latent class membership.

To further validate the multinomial logistic regression model, we highlight the distinct trends in Figure 3. As shown, materialism and preference for sexiness clearly differentiate the body-oriented classes from the body-unoriented class, with substantially higher logit coefficients for the former. This pattern aligns with theoretical expectations in that materialism entails a stronger valuation of visible status signals, including a partner's physical attractiveness, and a greater preference for sexiness corresponds to increased emphasis on bodily attractiveness. In contrast, egalitarianism and intellectual orientation are associated with lower logit values, reinforcing their alignment with the body-unoriented profile. This is theoretically meaningful, as egalitarian values tend to downplay status, whereas intellectual orientation places greater importance on personal qualities rather than physical qualities. That these predictors distinguish the latent classes in theoretically expected directions offers compelling evidence for the validity of the class solution, suggesting that the model has successfully uncovered meaningful and interpretable latent classes within the data.

### Consistently-Appearing Buttocks-Oriented Class

**Figure 4.**
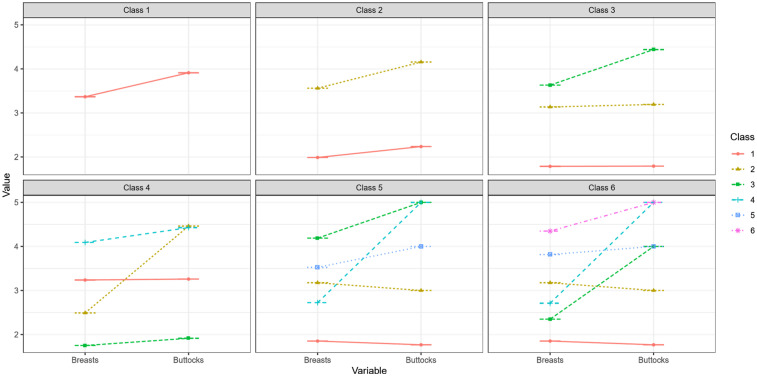
Depiction of the six latent class solutions.

As shown in Figure 4, a consistent pattern emerges across all six latent profile solutions: in each model, at least one class exhibits a clear preference for buttocks over breasts, as indicated by consistently higher mean ratings for buttocks. This recurring trend suggests the presence of a stable buttocks-oriented latent profile that persists regardless of the number of extracted classes. The repeated emergence of this class across varying model solutions provides strong evidence for the robustness and substantive distinctiveness of this profile within the data.

## Discussion

The primary aim of the present study was to investigate whether human males could be classified based on their preferences for two evolutionarily significant physical features: breasts and buttocks. Our findings indicate the existence of distinct subgroups within the Norwegian male population, as we identified four latent classes based on the extent to which these features were deemed important in the evaluation of romantic partners. The largest group, Highly Body-Oriented (53%), rated both breasts and buttocks as highly important. The second group, Moderately Body-Oriented (20%), also valued both features, though to a slightly lesser degree. The third group, labeled Buttocks-Oriented (18%), showed a clear preference for buttocks over breasts. Lastly, the Body-Unoriented group (9%) placed minimal importance on either feature in their partner preferences.

What do these findings reveal? While it is well-established that men place greater emphasis on physical features when evaluating potential mates (Buss, 1989; Mehmetoglu et al., 2024; Meltzer et al., 2014), we have lacked clarity regarding which specific features drive this preference. The current study provides compelling evidence that breasts and buttocks significantly shape men's perceptions of female physical attractiveness, doing so for over 90% of the Norwegian men in our sample. Interestingly, for approximately 10% of participants, these features appeared to have minimal influence. This suggests that a minority of men may prioritize other physical traits, or perhaps socio-psychological or cognitive characteristics. Future research should focus on examining and profiling this subgroup, which we identified as Body-Unoriented, to better understand the alternative criteria they consider important. Qualitative approaches, such as focus group interviews, could offer deeper insight into the mating psychology and preferences of this marginal group.

Equally interesting is that our study highlights a subtle yet important point: buttocks function as a more central cue than breasts in structuring male preference patterns in this sample. While the evolutionary psychology literature often gives the impression that these two features are equally valued, our findings challenge this assumption. Specifically, we identified a distinct subgroup of men (Buttocks-Oriented) who showed a marked preference for buttocks, but no corresponding subgroup that prioritized breasts exclusively. This asymmetry supports an earlier observation by Dagnino et al. (2012), who also reported a stronger preference for buttocks. One possible explanation is that buttocks may have had a longer evolutionary history in influencing male mating psychology, thereby leaving a deeper imprint on male preferences.

It is clear that although breasts are considered important in men's evaluations of female attractiveness, they consistently appear in conjunction with buttocks across all identified subgroups. This suggests that men value breasts, but primarily when paired with buttocks. Put more plainly, men may derive sufficient cues related to youth, fertility, nutritional status, and developmental stability by observing the buttocks alone. In this context, breasts appear to serve a complementary rather than primary role. These hypotheses warrant further investigation in future research. One approach could involve designing an experiment in which participants rate the attractiveness of female figures based on systematically varied combinations of breast and buttock images, differing in symmetry, size, curvature, shape, and other relevant features. Such a study could draw on insights from the individual work of Dixson et al. (2011) and Lewis et al. (2015).

Another promising direction for future research would be to examine whether theoretically grounded facial traits, such as hair and facial symmetry, are equally important in shaping male preferences. Researchers could apply LPA to examine whether distinct subgroups emerge based on ratings of facial features alone as well as on combinations of facial and bodily features. Furthermore, future studies could extend this approach by comparing not only specific physical features, such as buttocks versus breasts or hair versus facial symmetry, but also by contrasting broader categories, such as facial versus bodily traits, and physical versus non-physical traits (e.g., socio-cultural and cognitive attributes), to better understand their relative importance in human mate choice.

From a theoretical perspective, the external predictors indicate that the classes are not just statistical categorizations derived from responses about the importance of breasts and buttocks in partner selection. Rather, these preferences appear to be embedded in broader attitudinal and value orientations. Individuals who place greater emphasis on visible status and sexual attractiveness were more likely to belong to the body-oriented classes, whereas those endorsing egalitarian values were more likely to fall into the body-unoriented class. In other words, these overarching value orientations likely reflect underlying dispositions that give rise to the observed clustering of latent profiles in the present study. Importantly, when these predictors were entered simultaneously, they accounted for approximately 20% of the variation in class membership. In the context of multinomial logistic models, this represents a considerable proportion of explained variation and provides further support for the substantive relevance of the identified profiles.

Our study has a few limitations that should be considered when interpreting the results. However, these limitations also highlight potential avenues for future research. A key direction would be to replicate similar studies across diverse cultural contexts, including non-WEIRD societies, which could help refine or strengthen the generalizability of the current findings. Another notable limitation is the use of a convenience sample, which limits the extent to which the results can be generalized, despite the theoretical rationale supporting their plausibility. Future research could address this issue by employing more representative sampling strategies where feasible. A final limitation is that egalitarianism and materialism were assessed using single-item measures rather than validated multi-item scales. These variables were included as coarse external validators to test directional differentiation of the latent classes, not to provide comprehensive measurement of the constructs. Future research should replicate the findings using established multi-item instruments.
